# Using the Lives Saved Tool as part of evaluations of community case management programs

**DOI:** 10.7189/jogh.04.020412

**Published:** 2014-12

**Authors:** Ingrid K. Friberg, Neff Walker

**Affiliations:** Department of International Health, Johns Hopkins Bloomberg School of Public Health, Baltimore, MD, USA

## Abstract

**Background:**

Integrated community case management (iCCM) has been recommended by the World Health Organization to reduce mortality among children in populations with limited access to facility–based health care providers. Although many countries have introduced iCCM, interpretation of the impact is difficult due to many other activities occurring in the community. This paper suggests a method for using the Lives Saved Tool to model the independent impact of iCCM on child mortality.

**Model:**

The Lives Saved Tool (*LiST*) is a multi–cause model of mortality which allows users to look at the potential impacts of one or many interventions on one or many causes of death without double counting their impact. *LiST* uses changes in intervention coverage and cause–specific effectiveness estimates on mortality and risk factors to model overall changes in mortality as well as to attribute mortality reduction to specific interventions. Collecting data on the source of the care seeking behaviors is critical to being able to model and interpret the changes observed.

**Discussion:**

The complexity of implementation of iCCM in the environment of broader health changes requires modeling to understand the program specific impacts. Using *LiST* results as additional data in combination with observed coverage change and mortality reduction can help explain the isolated impact of a given iCCM program when other changes are ongoing. *LiST* is unable to determine why the changes in health care seeking behaviors occur, but can be useful in helping to explain whether or not the changes were beneficial.

Under–5 mortality is a continuing problem globally, with over 6.6 million children still dying annually [1]. In Africa, the most common causes of death include pneumonia, diarrhea, malaria and prematurity [2]. The World Health Organization published a position paper in 2012 promoting implementation of community case management of childhood illnesses to reach populations with limited access to facility–based health care providers with the aim of reducing child mortality [3]. Typically, integrated community case management (iCCM) includes antibiotics for pneumonia, oral rehydration salts and zinc for diarrhea and appropriate antimalarial drugs (artemesinin combination therapy, with or without use of rapid diagnostic tests, RDTs). Many countries have been expanding the role of community health workers to include iCCM [4-6] (ie, Ethiopia, Malawi). For further expansion, the likely impact of this new delivery strategy needs to be quantified and made available to potential implementers, funders and international organizations such as WHO and UNICEF [5,7].

Much of the data regarding changes in mortality come from two sources: controlled studies of implementation of a project [8,9] and large national surveys of mortality and health intervention coverage [10]. Both have limitations with respect to understanding the potential impact of a specific program on mortality rates. Controlled studies often only measure the exact program, not the wider environment while national surveys typically estimate population wide mortality rates, but do not necessarily know the source of the changes [1, 6]. In both types of data, the impact of the specific implementation program is confounded by fluctuations within the wider health care system, including stockouts of medication or supplies, nonfunctioning equipment, health care worker strikes, and weather events [11,12]. Given these possibilities, neither an increase nor a decrease in coverage of a specific intervention will be adequate to estimate the impact of a given health service delivery mechanism or program.

In addition, a fully randomized trial evaluating the impact of an iCCM program is not possible under most circumstances. Instead quasi–experimental designs are used, and within both the intervention and comparison districts, other child health interventions such as vaccination, nutrition programs may be changing. In these studies, one must estimate the impact of changes in all of the interventions, not just the ones provides by iCCM programs in evaluating the impact of the program. In response to this type of complexity, it has been argued that the use of modeling will play a critical role in making causal inferences linking programs and impact [13].

This paper describes a methodology for using the Lives Saved Tool (*LiST*) to model the impact of iCCM within the wider changes of health intervention coverage.

## METHODS

### Overview of the Lives Saved Tool

The Lives Saved Tool (*LiST*) [14] models the impact that increased coverage of health interventions will have on under–5 mortality [15], neonatal mortality [16], maternal mortality [17] and stillbirths [18,19]. It is situated within the Spectrum Policy Modelling Software and utilizes formal links to the AIDS Impact Module (AIM), the Family Planning Module (FamPlan) and the Demography Module (DemProj) [14,20]. It has been characterized as a linear, mathematical model that is deterministic. The fixed relationships between inputs and outputs will produce the same results each time one runs the model. The primary inputs are coverage of interventions while the outputs are changes in population levels of risk factors (such as wasting or stunting rates, birth outcomes such a prematurity or size at birth) and cause–specific mortality (neonatal, child mortality (1–59months), maternal mortality and stillbirths). The relationship between a given input (change in intervention coverage) and one or more outputs is specified in terms of the effectiveness of the intervention in reducing the probability of that outcome. The overarching assumption in *LiST* is that mortality rates and cause of death structure will not change except in response to changes in coverage of interventions or other proximate determinants. The model assumes that changes in distal variables such as increase in per capita income or mothers’ education will affect mortality by increasing coverage of interventions or reducing risk factors.

There are 68 separate interventions within *LiST*, affecting risk factors or causes of death; interventions can be linked to one or multiple outcomes. A key feature of *LiST* is that it allows one to look at the impact of scaling up coverage of multiple interventions simultaneously without double counting the impact, instead of only assessing a single intervention and a single cause of death as is done in many natural history models.

### Mortality reduction calculations with LiST

Several structural features of *LiST* must be considered in order to estimate the impact of scaling up coverage of multiple interventions and changes in risk factors on mortality. First, the effectiveness or efficacy of an intervention must be described in terms of reduction in cause–specific mortality rather than in overall mortality. With cause–specific estimates of effect, we can then compute the combined impact of interventions. When there is a single intervention, the calculation of impact is simple as one has change in coverage times the efficacy of the intervention and this impact is applied to the cause–specific mortality. For example, we may have population with 10 000 diarrhea deaths in children aged 1–59 months where we introduce a new vaccine that would be 50% effective in reducing diarrhea mortality. If we reach coverage of 50%, we would then reduce diarrhea deaths to 7500 (10 000 – [10 000 × 0.5 × 0.5]). With a second or a third intervention, the same approach is followed except that the second diarrhea intervention would only be applied to the residual un–prevented diarrhea deaths. If the second new diarrhea intervention is also 50% effective and coverage reaches 50% we would then reduce diarrhea mortality to 5626 (7500 – [7500 × 0.5 × 0.5]). By using cause–specific efficacy and applying each intervention to the residual deaths after we have estimated the impact of previous interventions, we ensure that we are not double counting the overall impact of interventions on mortality.

### Attribution of lives saved by intervention

Another output of the *LiST* model is an attribution of lives saved to changes in coverage of interventions and risk factors. When a single intervention is scaled up, attribution is simple. However, when multiple interventions acting on the same cause of death are scaled up, one must have a consistent approach to make the attribution. In *LiST,* attribution is applied first to all preventive interventions (sequentially across the continuum of care, from periconceptual, through pregnancy, delivery and then postnatal preventions), and subsequently to the treatment interventions. Thus, if both a preventive and a treatment intervention are scaled up, the full effect of change in coverage of the preventive intervention is calculated and attributed to the preventive intervention. Then the residual deaths averted are attributed to the treatment scale up. When there are two or more interventions either in preventive or treatment categories, there is a second step in the attribution calculation. First we compute the number of lives saved by applying all preventive interventions. Then the attribution is based on the proportional impact of the preventive interventions, calculated as the increase in coverage times the effectiveness of the intervention.

In addition to reporting the impacts or attributions associated with a specific set of data, *LiST* can also be used to compare results from multiple scenarios and assess differences of multiple options. The choice of the exact two scenarios to be compared determines the interpretation of the results.

### How LiST has been used

One of the primary ways in which *LiST* has been used is to help countries develop strategic plans for maternal and child health. One example of this type of work was the development of possible scale up scenarios for high mortality countries in sub–Saharan Africa [19]. In this analysis, *LiST* was used to estimate the impact of a small set of effective interventions which could be delivered. The analyses were used by countries to help set priorities in their efforts to reach their MDG goals.

*LiST* has also been used to help explain which programs or activities led to measured reductions in mortality. For example, in a recent analysis of Niger [21], the *LiST* model was used to help disambiguate a complex set of changes in coverage of many interventions at the national level that led to a 50% reduction in under–five mortality in a the past 10 years. This analysis showed that while there were many interventions that had some impact on under–five mortality, the majority of the effect was due to scale up on interventions for malaria as well as reductions in stunting and wasting rates.

## RESULTS

### Use of *LiST* in the evaluation of iCCM Programs

*LiST for modeling observed outcomes.* The use of *LiST* in the evaluation of iCCM programs can be seen as a combination of the two methods (mortality reduction and attribution) briefly described above.

The introduction of iCCM into a population is intended to be a new delivery mechanism for ensuring the appropriate case management of childhood illnesses. This is often assumed to be in addition to existing sources of medication in a community, which can include health facilities and pharmacies. A typical national level survey would report total coverage of treatment, and would not differentiate the results by source in a standard results table [6]. This could indicate that total coverage of an intervention increased over time (as in **Table 1**: total coverage). However, this result would not differentiate between the ‘ideal results’, here used to indicate an effective iCCM program which reaches those not already accessing care, and other possible results that are less easy to interpret. The ideal results would show no change in coverage delivered via non iCCM mechanisms and thus the full impact could be assumed to be linked to the introduction of iCCM (as in **Table 1**: ideal results). It is more likely that results similar to the ‘potential results’ are driving the change in coverage.

**Table 1:** Overall coverage of an intervention increased post introduction of community case management

		Ideal Results		Potential Results	
	Total coverage	Community coverage	Facility coverage	Community coverage	Facility coverage
Before	30%	0%	30%	0%	30%
After	40%	10%	30%	2%	38%

A *LiST* analysis can be done with one additional piece of information from the survey – the source of the treatment to differentiate between these two options. With this additional question, *LiST* can quantify the impact, by comparing the total coverage changes observed over time to the total coverage delivered within facilities. Importantly, the total coverage of the intervention at baseline (before iCCM implementation; 30% in **Table 1**) modeled must be the same in the two comparison scenarios. The difference between these two scenarios (total coverage and observed facility coverage) would account for the impact of the community program. This is predicated on the assumption that the effectiveness of the intervention is the same given all possible delivery mechanisms.

*LiST for modeling hypothetical outcomes.* It is a more difficult situation to interpret when changes in the health system have occurred simultaneous to and independent of the introduction of the iCCM program. **Table 2** shows an example where total coverage has decreased over time, yet the apparent impact of the community delivery strategy is positive.

**Table 2:** Overall coverage decreased in the after of a before and after study design

	Total coverage	Community coverage	Facility coverage	Comparison scenario
Before	52%	5%	47%	5%*+47% = 52%
After	48%	15%	33%	5%*+33% = 38%

*The community coverage values at baseline and assuming no change over time.

**Figure 1:**
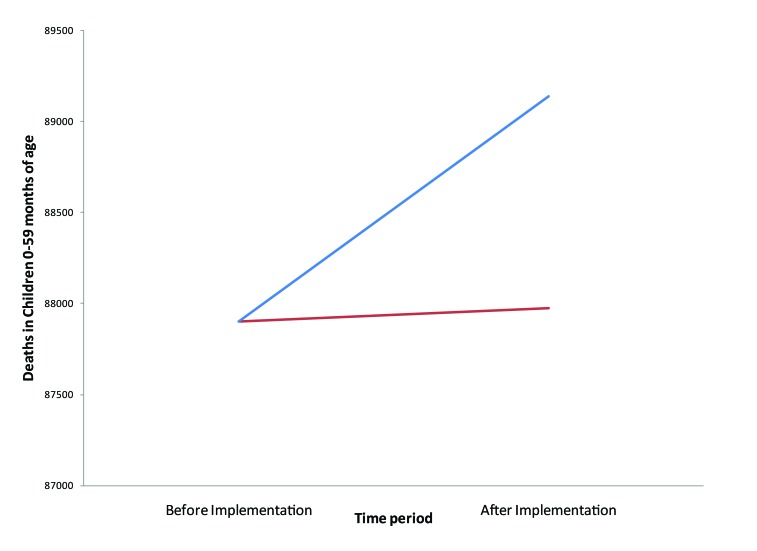
Predicted deaths modeled by *LiST*; observed data and hypothetical without community programming; based on data in **Table 2**. Red line – Scenario 1: predicted deaths using observed coverage changes, blue line – Scenario 2: predicted deaths using hypothetical coverage change, excluding community delivery.

Using *LiST* and the information on the delivery strategy can help to identify the impact of the program. In this situation, the two scenarios to be compared are the same as the previous example. The first scenario will show the overall coverage, while the second will show the coverage expected if there were no change in the community interventions. The difference between these scenarios shows the impact of the community case management intervention. In both scenarios, additional deaths relative to baseline will be observed due to both a reduction in coverage and an assumed increase in population (**Figure 1**). The observed total situation based on data in **Table 2**, will be modeled by the red line in **Figure 1**, while the hypothetical line for what would have happened without the community based portion of coverage change is the blue line. The difference between these two scenarios is the impact of the community program. If the deaths modeled by assuming no change in the community programming are greater than those observed, then the community program is having a positive impact regardless of the fact that the overall coverage is worse and overall deaths are increasing.

*LiST for modeling concomitant non–iCCM interventions.* Another key feature of *LiST* is the ability to distinguish the separate impacts of multiple interventions which are affecting a specific cause of death. One example is when assessing the impact of iCCM in communities where insecticide treated bednets (ITNs) are being rolled out simultaneously. A decline in malaria mortality will be observed regardless of the introduction of iCCM (**Table 3**). *LiST* can also determine which portion of the decline is likely due to iCCM and which is due to the ITNs. *LiST* results show that in the presence of ITNs (a prevention) the impact of antimalarials (a cure) will be smaller than when no ITNs are being deployed (2.9% vs 2.1% reduction in **Table 3**), indicating the critical nature of understanding the wider environment when assessing new programs. Similarly, it can distinguish if part of the overall mortality reduction is due to a change in the underlying prevalence of HIV within a community (not shown).

**Table 3:** Percent reduction in mortality by iCCM alone vs with ITNs

	ITNs alone	iCCM alone	iCCM and ITNs
ITNs	3.7	–	3.7
Antimalarials	–	2.9	2.1
Case management of pneumonia	–	2.7	2.7
ORS + Zinc for diarrhea	–	2.4	2.4
Total Percent Mortality Reduction	3.7	7.9	10.9

iCCM – integrated community case management, ITN – insecticide treated bednets, ORS – oral rehydration salts.

## DISCUSSION

The Lives Saved Tool is a multi–cause model of mortality which allows users to compare observed data with hypothetical comparison information. It allows users to isolate the impact of a particular program when looking at the observed mortality changes and coverage changes in a population. Integrated community case management is a new delivery mechanism which has the potential to reduce inequities by reaching the most marginalized within a community. *LiST* can easily compare the difference between the observed situation and the hypothetical where iCCM did not exist. This is especially important in areas where external events confuse the overall impacts of a program, for example stockouts or strikes by facility workers. *LiST* does not have the ability to understand why the observed changes are occurring. However, the user can explicitly state the assumptions of what would likely have occurred without the new program. This is an advantage when trying to interpret mortality rates which may be unchanged or increase in the study area and or the comparison area.

The real world experience with iCCM has been difficult to interpret because the interventions that are part of iCCM are not being delivered in a vacuum. Other interventions may have also been scaled up and these interventions, not the iCCM, may drive measured mortality reduction. Additional issues such as stockouts at the facility level could result in people shifting to using community health workers, simply because stock was available in the community and not the facility. The reality may be that there are no additional people seeking care from the community health worker. Another issue may be task shifting. People who are already seeking care simply prefer the convenience of the community workers and choose to use them instead of the facility workers. In both of these situations, assessing the utilization of the community health workers does not tell the complete story of the impact of iCCM. It is necessary to further describe the changes in total coverage of health interventions in order to understand whether or not a mortality benefit should be attributed to the new program.

It should also be noted that *LiST*, which focuses on mortality, only generates one type of data for understanding the impacts of any health program. These results should be used in combination with other data types, such as qualitative and quantitative data on program users as well as the costs of implementation, among others. Together, a broader understanding of total impact can inform all aspects of the relevant discussions on whether expansion is warranted and benefits are being accrued. This paper has extensively discussed the benefits of using *LiST* as one tool within an evaluation toolkit within iCCM. It can also be used prospectively to help identify what potential impacts an iCCM program could expect if a new program were implemented in a specific country or region. This can help the health programmers tailor the program correctly, in terms of focus, methods and location, as well as to understand what competing interventions would be critical to understand. This would help to ensure that all prospective survey data that were relevant were collected.

Using modeling to evaluate program specific mortality results has several limitations. First, comprehensive data needs to be collected prospectively with an eye on the modeling needs. Those collecting survey data need to capture all information relevant to the causes of death of interest, even if they are not program specific interventions. This may make it more difficult to consider modeling retrospectively, which is the typical experience currently. It may also limit the ability to correctly interpret the predicted mortality rates due to the many unknowns, which may be very expensive and time consuming to collect. In addition, it is critical that one consider the quality of the data. The poorer the quality as well as the sparser the data, the less likely that meaningful results can be derived from modeling. A current additional limitation is the lack of empirical data quantifying the difference in effectiveness between delivery points for the iCCM interventions. There are likely to be differences in both the population receiving care by different providers and at different locations as well as in how effective the intervention is going to be when delivered by those providers. These combine to result in the true impact differences being greater or smaller than expected with the single effect size currently available. Studies of iCCM implementation may also be completed in atypical environments, which limit their generalizability while at the same time overall population based mortality data cannot answer the question about whether or not iCCM is driving the observed changes. The observed implementation data are often confounded by fluctuations within the wider health care system which are not under the control of an implementer. Thus, neither an increase nor a decrease in coverage of a specific intervention will be adequate to estimate the impact of a given health service delivery mechanism or program. The use of a modeling tool such as *LiST* can help to tease out the impacts which can be attributed to a given program. These data can be used by implementers, funders and international organizations as they discuss the merits of initiating an iCCM program in a particular community.
